# Omnidirectional Walking Pattern Generator Combining Virtual Constraints and Preview Control for Humanoid Robots

**DOI:** 10.3389/frobt.2021.660004

**Published:** 2021-06-01

**Authors:** Francesco Ruscelli, Arturo Laurenzi, Enrico Mingo Hoffman, Nikos G. Tsagarakis

**Affiliations:** Humanoids and Human Centered Mechatronics (HHCM), Istituto Italiano di Tecnologia (IIT), Genova, Italy

**Keywords:** walking pattern generation, bipedal locomotion, humanoid robots, whole-body control, motion control

## Abstract

This paper presents a novel omnidirectional walking pattern generator for bipedal locomotion combining two structurally different approaches based on the virtual constraints and the preview control theories to generate a flexible gait that can be modified on-line. The proposed strategy synchronizes the displacement of the robot along the two planes of walking: the zero moment point based preview control is responsible for the lateral component of the gait, while the sagittal motion is generated by a more dynamical approach based on virtual constraints. The resulting algorithm is characterized by a low computational complexity and high flexibility, requisite for a successful deployment to humanoid robots operating in real world scenarios. This solution is motivated by observations in biomechanics showing how during a nominal gait the dynamic motion of the human walk is mainly generated along the sagittal plane. We describe the implementation of the algorithm and we detail the strategy chosen to enable omnidirectionality and on-line gait tuning. Finally, we validate our strategy through simulation experiments using the COMAN + platform, an adult size humanoid robot developed at Istituto Italiano di Tecnologia. Finally, the hybrid walking pattern generator is implemented on real hardware, demonstrating promising results: the WPG trajectories results in open-loop stable walking in the absence of external disturbances.

## 1 Introduction

**FIGURE 1:**
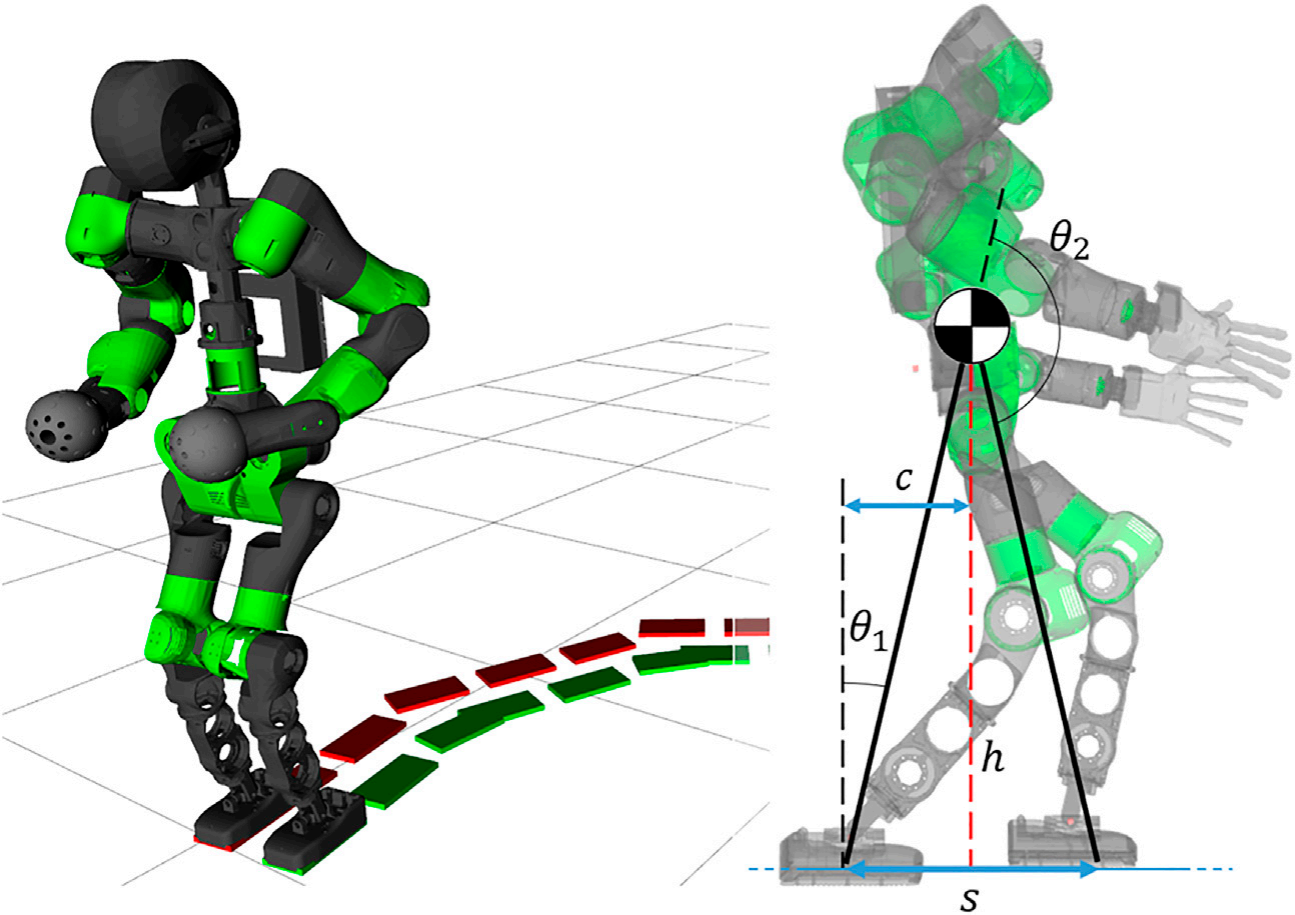
Left: the humanoid robot COMAN + walking while changing direction. Right: the template model for the VC, the compass walker, superimposed on the robot kinematics.

Humanoid walking has improved greatly in the last years as many strategies were introduced by the research community. However, controlling bipedal robots is still a challenging task due to the gap between simulation and mechanical hardware, mainly embodied by model inaccuracies, the intrinsic complexity of the humanoid platform, the inherent instability of the system and the challenging estimation of the floating base pose. The *Zero Moment Point* (ZMP) criterion (Vukobratović and Borovac, 2004) is still widely used for locomotion, thanks to its applicability to diverse robots and gaits: however, it guarantees a stable motion by While being a less restrictive constraint than walking without violating *quasi-static stability* (the ZMP is restricted inside the support polygon, but the CoM can travel beyond), the resulting motion is unnatural and under-achieving in terms of efficiency compared to the human walk, which, on the contrary, entails a portion of the stepping motion where the ZMP lies on the edge of the support polygon. Exploiting a different notion of stability, which relies on limit cycles, can produce more dynamical and efficient gaits: however, its practical limitations reside in the extreme dependence on the accuracy of the model and the gait design restrictions that only allows periodic motions, often with a small basin of attraction. It was shown in biomechanics that the dynamical motion is generated for the most part by a controlled fall in the forward direction: the ZMP travels along the sagittal plane reaching the corresponding edge of the support polygon, while in the lateral direction it remains well inside the support region (Sardain and Bessonnet, 2004). The proposed walking pattern generator (WPG) is based on the limit cycle theory, which aims at producing a cyclic period-one gait. In doing so, we keep the paradigm formulated in (Ruscelli et al., 2019): in the sagittal direction, where the dynamical component of the gait is mainly found, we exploit *Virtual Constraints* (VC) to design a template periodic motion. Along the lateral plane, we rely on *Preview Control* (PC): by treating the robot as a one-dimensional linear inverted pendulum model (LIPM), the input-output relationship between CoM and ZMP can be exploited to compute a CoM trajectory to track a feasible sideways ZMP reference which satisfies the ZMP stability criterion. While gait generation using the well-known PC-based WPG is a solved problem in literature, the proposed hybrid algorithm is not trivially generalizable to an omnidirectional walk. In this work, we present an upgraded version of our hybrid WPG including the following contributions:

• we enhance the versatility of the gait, allowing to change heading, step length and feet distance;
• we enable user inputs to modify on-line the gait.
  These high-level improvements involve substantial changes in the two components of the framework and their interaction:
• *Sagittal plane*: footsteps are no more planned beforehand, but automatically generated on-line given the maximum inclination the angle of curvature;
• *Lateral plane*: the preview window is continuously updated with a suitable ZMP reference to accommodate the gait;
• *Synchronization*: the algorithm to synchronize the sagittal and lateral plane is redesigned to allow omnidirectionality;

The algorithm results in a light-weight, omnidirectional WPG^1^ based on a simple template model that can be easily deployed on any humanoid robot (as shown in Figure 1). This method allows for a more dynamic motion without dropping the advantages of the PC and the ZMP criterion. Furthermore, redesigning the strategy for synchronization not only allows to drop a heuristic approach, but provides for the implementation of omnidirectionality in the WPG. The template model is mapped on the robot using the whole-body inverse kinematics (IK) solver by specifying a set of desired Cartesian tasks sorted by priority, so as to impose a desired body posture besides directly controlling the model variables as high priority tasks, i.e., the feet trajectories and the composite motion of the sagittal and the lateral components of the CoM. Finally, we discuss the advantages and the limitations of this approach and we propose future improvements.

## 2 Related Works

The first paradigm formulated for humanoid walking is known as *static stability*, which restricts the projection on the ground of the center of mass (CoM) to lay inside the support polygon at all times. In this way, a *static walk* can be simply synthesized by slowly moving the CoM from one foot to another (Kato et al., 1974). The seminal work of Kajita (Kajita et al., 2003) demonstrated how, using the *Preview Control* theory and leveraging on the notion of the ZMP, a more dynamic, stable gait can be achieved. The effectiveness of this approach made it widespread among the humanoid locomotion community and various improvement were proposed: the LIPM model was extended to reduce the modeling error and generate a robust pattern locomotion (Shimmyo et al., 2013). Application that enhance the preview control reacting against external perturbations (Nishiwaki and Kagami, 2006; Nishiwaki and Kagami, 2009) or adapting to uneven terrains (Kajita et al., 2006). The simple yet successful strategy is usually framed into an *Model Predictive Control* (MPC) formulation, which is still the most widely adopted: a method to guarantee its intrinsic stability was presented in Scianca et al. (2016), and it was extended for disturbances rejection (Wieber, 2006), push recovery (Stephens and Atkeson, 2010) and automatic footstep placement (Herdt et al., 2010). The new strategy which released the constraints of the ZMP by introducing a different notion of stability based on limit cycles was first proposed in Hürmüzlü and Moskowitz (1986). The theory was further developed in Westervelt et al. (2007) using *Hybrid Zero Dynamics* (HZD) to actively control the humanoid and enlarge the basin of attraction of a limit cycle gait. This approach yielded successful results, but it is usually limited to robots with particular body structures (Rezazadeh et al., 2015; Gong et al., 2019). Some strategies were explored to generalize this method to the 3D case, such as the functional Routhian reduction introduced in Chevallereau et al. (2014), or the self-synchronization exploiting the symmetry of the system, formalized in Razavi et al. (2015). In fact, while producing dynamical and energy-efficient gaits, this method is not trivially generalized to the 3D case, where hybrid invariant manifolds are more challenging to describe. Nonetheless, some techniques were developed to implement 3D walking on different robots (Da et al., 2016; Reher et al., 2016), most of them decoupling the walking gait into forward and lateral motion. Furthermore, most of the strategies solely consider straight walking gaits, as steering cannot be trivially integrated. While some elegant solution can be found in literature, they are mostly theoretical results, usually verified only in simulation (Motahar et al., 2017; Chevallereau et al., 2010).

The proposed hybrid WPG relies on the synchronization of the sagittal and lateral planes of the robot, as in the previous works. However, it merges results from the VC and the PC theory: it exploits a more dynamical approach that only constrains the ZMP trajectory to the support polygon along the lateral direction. Furthermore, as opposed to the classical implementation of the VC, our algorithm does not heavily depend on the kinematics of the robot and does not require burdensome parameter tuning: template trajectories can be designed and realized on-line on the real robot, making it a computationally lightweight and flexible tool independent of the structure of the walker.

## 3 Sagittal Plane: Virtual Constraints

Virtual constraints are employed to combine the kinematics of many joints into a single template motion. This simplifies their coordination and reduces the dimension of the problem: the desired motion for a *n*-dimensional system can be encoded by controlling a smaller set of state variables *n−n*_*m*_, where *n*_*m*_ is the number of constrained variables. On the other hand, its inherent limitation resides in the cyclic nature of this template movement, which evolves along a periodic orbit. While VCs are useful for the generation of repetitive gaits, they are unsuited for any action that alters their evolution, such as turning steps or aperiodic manoeuvres.

**FIGURE 2:**
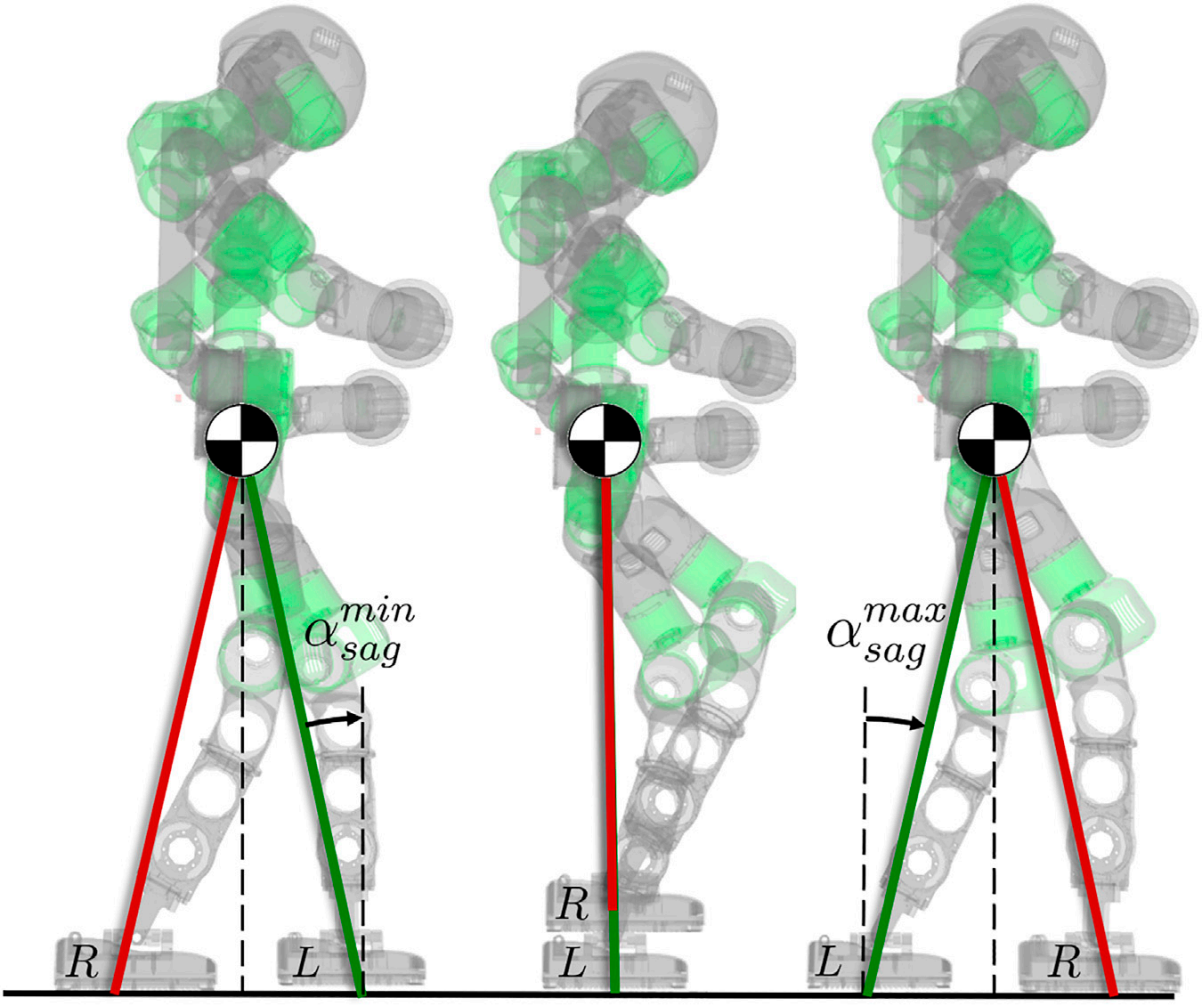
Evolution of one stepping cycle of the right leg from taking off to landing. The 2-link compass model is used as a template to impose the desired VC on the robot. The phase variable $\alpha_{sag}$ increases from $\alpha_{sag}^{min}$ to $\alpha_{sag}^{max}$ and constrains the movement of the CoM and the swing foot in the sagittal direction. The VC always constrains the swing leg to mirror the stance leg.

To illustrate this, let $x\in {\mathbb{R}}^{n}$ be the state vector of a *n*-link bipedal mechanism and $x_{c}:=h:{\mathbb{R}}^{n}\to {\mathbb{R}}^{m}$ a collection of *m* internal variables bound together by the VCs $h\left(x_{c}\right)$, to encode a specific locomotion. One VC is imposed on the mechanical system by zeroing the output of the function:

$$z=h\left(x_{c}\right)-h_{d}\left(\alpha \left(x_{f}\right)\right),$$ (1)

so that $h\left(x_{c}\right)$ evolves along the periodic orbit *h*_*d*_ as function of $\alpha \left(x_{f}\right)$, known as *phase variable*. The phase variable, in turn, is defined as a function of a set of *r* variables $x_{f}:{\mathbb{R}}^{n}\to {\mathbb{R}}^{r}$, which, oppositely to the controlled variables, remains free. While $\alpha \left(x_{f}\right)$ monotonically increases from $\alpha^{min}$ to $\alpha^{max}$, the controlled variables $h\left(x_{c}\right)$ are forced along a desired trajectory that translates into a specific stepping motion, as shown in Figure 2. Usually the output in (1) is zeroed by a feedback controller, which asymptotically brings the state variables $h\left(x_{c}\right)$ towards the desired orbit *h*_*d*_. In the work (Westervelt et al., 2007) is shown how suitable VCs can produce cyclic motions for a walker: the robot is treated as a point-foot template model, and the evolution of the internal joints is constrained as a function of the angle between the ground and the virtual leg connecting the stance foot and the CoM. Our strategy is based on the same formulation, which we detail in the following paragraph.

### 3.1 Template-Space Constraints

The template model of the humanoid robot is the 2-link compass walker, with its CoM located at the joint connecting the two links of equal length, as shown in Figure 1. The configuration vector is $\theta ={\left[\theta_{1},\theta_{2}\right]}^{T}$, where $\theta_{1}$ and $\theta_{2}$ are the angles describing respectively the *stance* and *swing* leg. The selected VC exploits the symmetry of the model by forcing the swing leg to behave as a mirror image of the stance leg. Its formulation for the compass geometry is the following:

$$z=\theta_{2}-\pi +2\theta_{1}.$$ (2)

According to (2), $\theta_{1}$ is selected as the phase variable $\alpha \left(x_{f}\right)$, while $\theta_{2}$ is the controlled variable constrained to the evolution of $\theta_{1}$. Finally, *z* is the output to be driven to zero.

### 3.2 Task-Space Constraints

The control of our humanoid robot relies on a whole-body hierarchical inverse kinematics framework: multiple Cartesian tasks can be defined and organized in a *Stack of Tasks* (SoT) fashion. Therefore, instead of defining the VC in the joint-space, we describe it as a function of Cartesian variables. Similarly to the formulation in the template-space, we are interested in binding relevant quantities of the robot (i.e., the CoM and the feet) with the phase variable α^2^. In the real robot, we choose α as the angle between the normal of the ground and the virtual leg connecting the CoM to the stance foot, as shown in Figure 1, which corresponds to the *tilt angle* $\theta_{1}$. As $\theta_{1}$ increases, the robot tilts on his stance foot in the sagittal direction, resulting in a CoM displacement *c*:

$$c=h\;\text{tan}\left(\theta_{1}\right),$$ (3)

where *h* is the height of the CoM w.r.t. the ground. By formulating the step displacement *s* as function of the configuration vector θ we obtain:

$$s=h\;\text{tan}\left(\theta_{1}\right)+h\;\text{tan}\left(\pi -\theta_{1}-\theta_{2}\right).$$ (4)

Finally, substituting $\theta_{2}$ in (2), yields *s* as a function of the *tilt angle* $\theta_{1}$ only:

$$s=2h\;\text{tan}\left(\theta_{1}\right).$$ (5)

This VC restricts the sagittal displacement of the swing foot to keep the CoM at a fixed distance from each leg: when the robot tilts forwards, the phase variable α increases, constraining the swing foot to perform a stepping motion until it impacts the ground, resetting the cycle. The VC does not constrain the trajectory of the leg, but only its overall displacement: thus, it can be treated as a task for the whole-body IK solver. This allow to tune the parameters of the stepping: We design the motion as a polynomial primitive with a peak of a desired height, which corresponds to the step clearance.

Following the theory in (Westervelt et al., 2007), the template model assumes fully inelastic collision, i.e. the landing foot does not experience slips or bounces at the contact point. For this reason, we impose zero-velocity of the swing leg at the beginning and at the end of the step, to reduce high impacts resulting in undesired rebounds of the foot on the ground that could disrupt the walking cycle. Finally, the impact is instantaneous: the stance leg is lifted at the same time the swing foot touches the ground, without any double stance phase during the gait.

### 3.3 Ankle Actuation

Once a suitable VC is applied, the template model can be treated as a simplified system with only one DoF, the *tilt angle* $\theta_{1}$. Its evolution, if not controlled, injects the free-fall dynamics of the stance leg, modeled as a linear inverted pendulum (LIP), into the system. This free evolution exploits the natural dynamics to reduce energy consumption during the stepping motion, but limits the flexibility of the gait, which is bound to a non-controlled variable. The feet of our humanoid are composed of flat soles and fully actuated ankles. Differently from the point feet of the template model, this allows to directly control the *tilt angle* $\theta_{1}$ of the robot and, as a consequence, the phase variable α. The CoM can be fully controlled as long as the ZMP remains inside the support polygon (the implications of this choice are discussed in Appendix). When on the edge, the robot becomes underactuated and starts tilting with its natural dynamics. Explicitly controlling the sagittal tilt precludes the possibility to exploit the natural dynamics of the robot in the forward motion to reduce the power consumption of the gait. On the other hand, dealing with a fully controllable system is advantageous, since it can be easily regulated to impose a desired behaviour. For instance, when steering, changing the feet distance or increasing the step stride. Finally, by fully controlling the CoM, it is possible to define a consistent system along the two planes to guarantee that the lateral plane can rely on the LIPM: without violating the Cartesian constraints between the sagittal displacement of the CoM and the swing leg, we impose a constant height to the CoM trajectory, realized by the HIK solver, so that the corresponding system in the lateral plane can be modeled as a LIPM.

## 4 Lateral Plane: Preview Control

The strategy exploited for the sagittal plane is simple to define in the planar case and allows a more dynamical motion, but it is not trivial to extend for 3D robots. Furthermore, the work in (Sardain and Bessonnet, 2004) demonstrates how the dynamic component of the walking is found along the sagittal plane of the foot: during a nominal gait, the ZMP travels mainly in the sagittal direction until it reaches the edge of the foot, without moving considerably along the lateral axis. For these reasons, we choose a conservative approach, as we rely on the well-grounded theory of preview control (Kajita et al., 2003): by treating the robot as a LIPM, we can impose a desired ZMP and track it by controlling the trajectory of the CoM.

In particular, the cart-table model describes a dependency between the projection along the lateral plane of the CoM $y_{\text{com}}\in \mathbb{R}$ and the ZMP $y_{\text{zmp}}\in \mathbb{R}$:

$$y_{\text{zmp}}=y_{\text{com}}-\frac{h_{\text{com}}}{g}{\ddot{y}}_{\text{com}},$$ (6)

where $h_{\text{com}}\in {\mathbb{R}}^{+}$ is the CoM height w. r. t the ground and *g* is the magnitude of the gravity vector.

Eqn. 6 is used to formulate the desired output-tracking problem. A common choice is to select the state vector $x\in {\mathbb{R}}^{3}$ as:

$$x={\left[\begin{array}{ccc} y_{\text{com}} & {\dot{y}}_{\text{com}} & {\ddot{y}}_{\text{com}} \end{array}\right]}^{T},$$ (7)

while setting the control input u∈ℝ as the lateral CoM jerk $\overset{\ldots }{y}$. By discretizing *via* zero-order-hold, We obtain a discrete-time LTI system, with time step *T*:

$$\{\begin{array}{cc} x_{t+1}=Ax_{t}+Bu_{t} & \\ y_{\text{zmp},t}=C_{\text{zmp}}x_{t} & \end{array},$$ (8)

where

$$A=\left[\begin{array}{ccc} 1 & T & T^{2}/2\\ 0 & 1 & T\\ 0 & 0 & 1 \end{array}\right],\;B=\left[\begin{array}{c} T^{3}/6\\ T^{2}/2\\ T \end{array}\right],$$ (9)

and the output, according to (6) is:

$$C_{\text{zmp}}=\left[\begin{array}{ccc} 1 & 0 & -\frac{h_{\text{com}}}{g} \end{array}\right].$$ (10)

As shown in (Kajita et al., 2003), the solution to this output tracking problem requires knowledge of the desired ZMP over a finite future horizon. We realize the anticipative action by framing the problem as a finite-horizon LQR. In particular, the optimal controller is formulated as follow:

$$\begin{array}{l} \underset{X,U}{\min}\overset{t+N}{\underset{k=t+1}{\sum }}{\left|\left|C_{\text{zmp}}x_{k}-y_{\text{zmp},k}^{\text{ref}}\right|\right|}^{2}+r\;{\left\|u_{k}\right\|}^{2}\\ \text{s}.\text{t}.\\ x_{k+1}=Ax_{k}+Bu_{k},\;k\in \left\{t,\ldots ,t+N-1\right\}, \end{array}$$ (11)

where $y_{\text{zmp},k}^{\text{ref}}$ is the desired ZMP at the *k*th future time step, and *r* > 0 is a weight that penalizes the control action. The QP problem (11) consists in minimizing the state $X={\left[x_{t+1},\ldots ,x_{t+N}\right]}^{T}\in {\mathbb{R}}^{3N}$ and the input trajectory $U=\left[u_{t},\ldots ,u_{t+N-1}\right]\in {\mathbb{R}}^{N}$ over the horizon of length *N* > 0, constrained to the dynamics of the system (8).

A solution is found *via* the *KKT equations*, which take the form of a highly sparse set of *n* = 4*N* + 3*N* equations in as many unknowns, solved through a sparse LU decomposition from the *Eigen3* library. Finally, at each iteration the first control input *u*_*t*_ is applied to the system (8); the resulting control law is linearly dependent on the current state *x*_*t*_ and the future reference ZMP trajectory $Y_{\text{zmp}}^{\text{ref}}\in {\mathbb{R}}^{N}$:

$$u_{t}=K_{\text{fb}}x_{t}+K_{\text{ff}}Y_{\text{zmp}}^{\text{ref}},$$ (12)

where $K_{\text{fb}}\in {\mathbb{R}}^{1\times 3}$ is a state feedback matrix, whereas $K_{\text{ff}}\in {\mathbb{R}}^{1\times N}$ is a reference feed-forward matrix. The state vector *x* is not updated with the real state of the robot, hence its future value *x*_*t*+1_ is obtained solely by integrating the current $x_{t}$ over a chosen period *T*, subject to the constant control input *u*_*t*_. The length *N* of the prediction horizon affects the tracking of the ZMP: a high *N* slows down the computation without greatly improving the performance, while a low *N* generates overshoots in the ZMP tracking. The system is kept stable thanks to the feedback term, hence computing the closed-loop eigenvalues magnitude is useful to assess the stability of the system:

$$\left|\lambda \right|<1\;\forall \lambda \in \text{sp}\left(A+BK_{\text{fb}}\right),$$ (13)

if the stability criterion (13) is not met, more of the future ZMP reference should be fed to the controller, increasing the length of the preview horizon, while ensuring that the resulting computational burden introduced by the length of the horizon can be handled within one control cycle of the system.

## 5 Synchronization

During a nominal walk, the motions along the two planes are strictly coupled together, since a sagittal displacement corresponds to a lateral movement of the CoM. In our previous work (Ruscelli et al., 2019), a strategy for synchronization between the lateral plane and the sagittal plane was proposed, showing how two structurally different control strategies can generate feasible references for walking. However, while being a viable method, it implied jumps or temporal freezes of the receding preview window: while the sagittal motion depended on the phase variable α, the ZMP window was dependent on time, and was reset at each step to synchronize with the stepping motion. Furthermore, it presented significant limitations for the generation of the future ZMP, as the receding window had a fixed ZMP pattern with predetermined values for the left and right foot. In this work, we propose a novel method for the synchronization of the planes. In the remaining of this Section, we outline the behaviour of the two independent component and we illustrate how they are coupled.

*Sagittal plane.* The sagittal displacement is dependent on the phase variable $\alpha_{sag}$ according to (3) and (5). A full cycle, from the take off of the swing foot to its landing on the ground entails $\alpha_{sag}$ increasing monotonically from $\alpha_{sag}^{min}$ to $\alpha_{sag}^{max}$, as shown in Figure 2.

**FIGURE 3:**
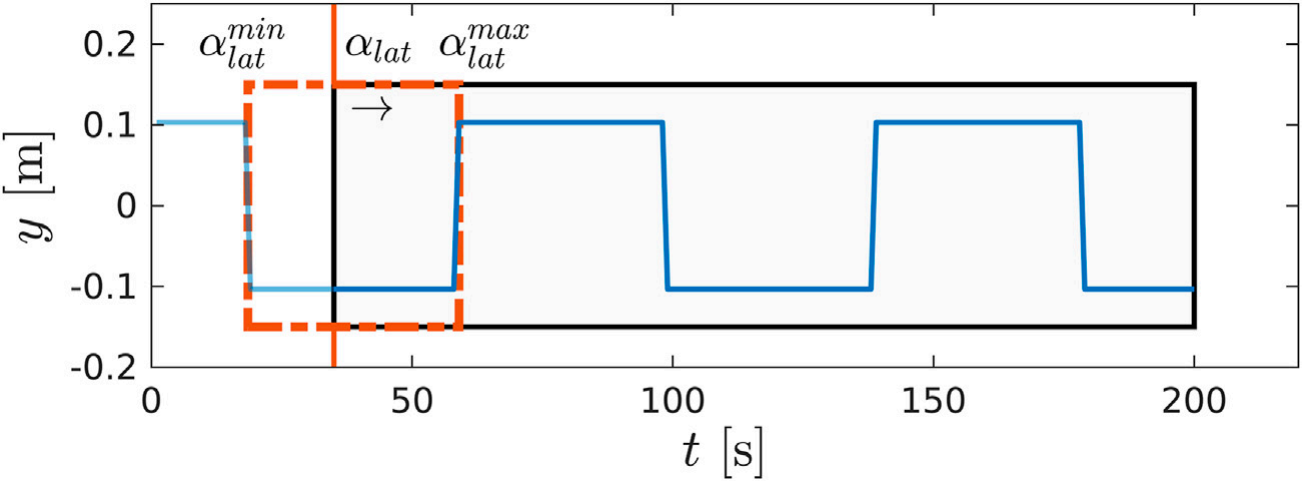
A snapshot of the preview window (in black) and the ZMP future reference (in blue) during a step. The advancement of the preview window does not depend on time, but it is bound to the evolution of the phase variable $\alpha_{lat}$ from $\alpha_{lat}^{min}$ to $\alpha_{lat}^{max}$.

*Lateral plane.* One complete cycle in the lateral plane corresponds to the advancement of the preview window: the ZMP reference resides inside the sole of the stance foot until the impact with the ground, when it switches to the opposite leg. We remove the dependency of the preview window on time and bind its advancement to the phase variable $\alpha_{lat}$. In particular, in one cycle the receding window slides from $\alpha_{lat}^{min}$ to $\alpha_{lat}^{max}$, as shown in Figure 3. The WPG automatically generates the values of the ZMP reference: the first segment of the preview window is the y-component of the position vector of the current stance foot, while the second segment is the y-component of the goal position of the swing foot. The remaining tail of the preview window is filled with a repeating pattern based on the ZMP values of the current and next steps.

**FIGURE 4:**
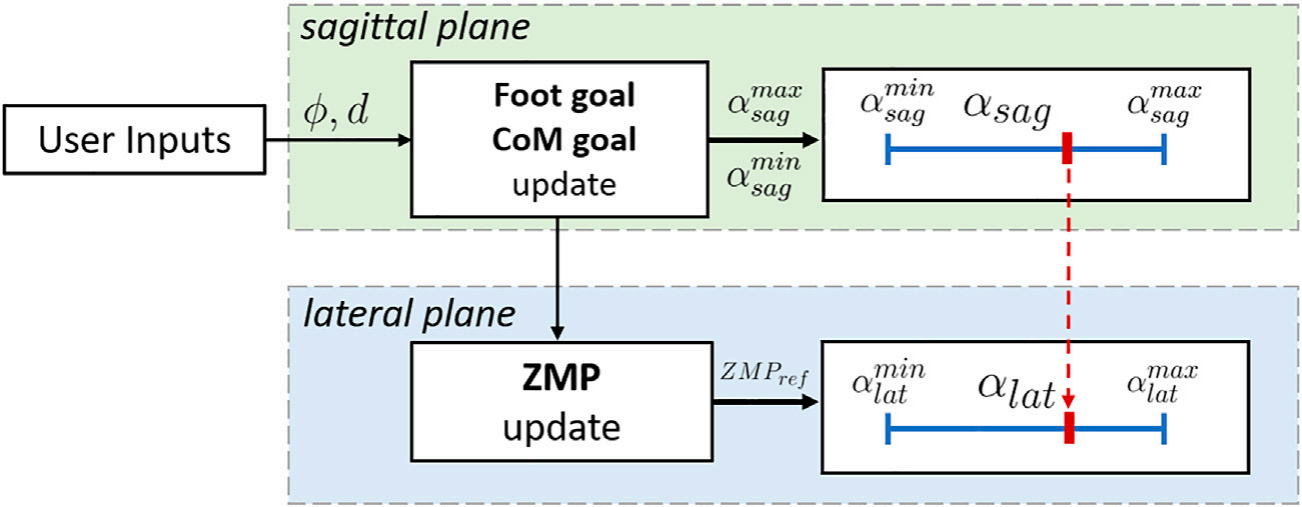
Detail of the synchronization process between lateral and sagittal plane. The commands θ and *d*, when received, update the foot and the CoM goal in the sagittal direction, which in turns modify the extrema of the phase variable, $\alpha_{sag}^{max}$ and $\alpha_{sag}^{min}$. The ZMP is updated accordingly, and used in the preview window fed to the PC. The lateral phase variable $\alpha_{lat}$ is synchronized with the evolution of $\alpha_{sag}$.

*Synchronization.* The phase variable $\alpha_{sag}$ is measured at each iteration, and its evolution coupled with $\alpha_{lat}$. Specifically, $\alpha_{lat}$ is directly driven by $\alpha_{sag}$, as its progression coincides with the monotonic increment of the sagittal phase variable, as depicted in Figure 4: the two planes are automatically synchronized, and the advancement of the CoM and the swing foot in the sagittal direction drives the lateral swing of the CoM along the full stepping cycle.

## 6 Implementation

**FIGURE 5:**
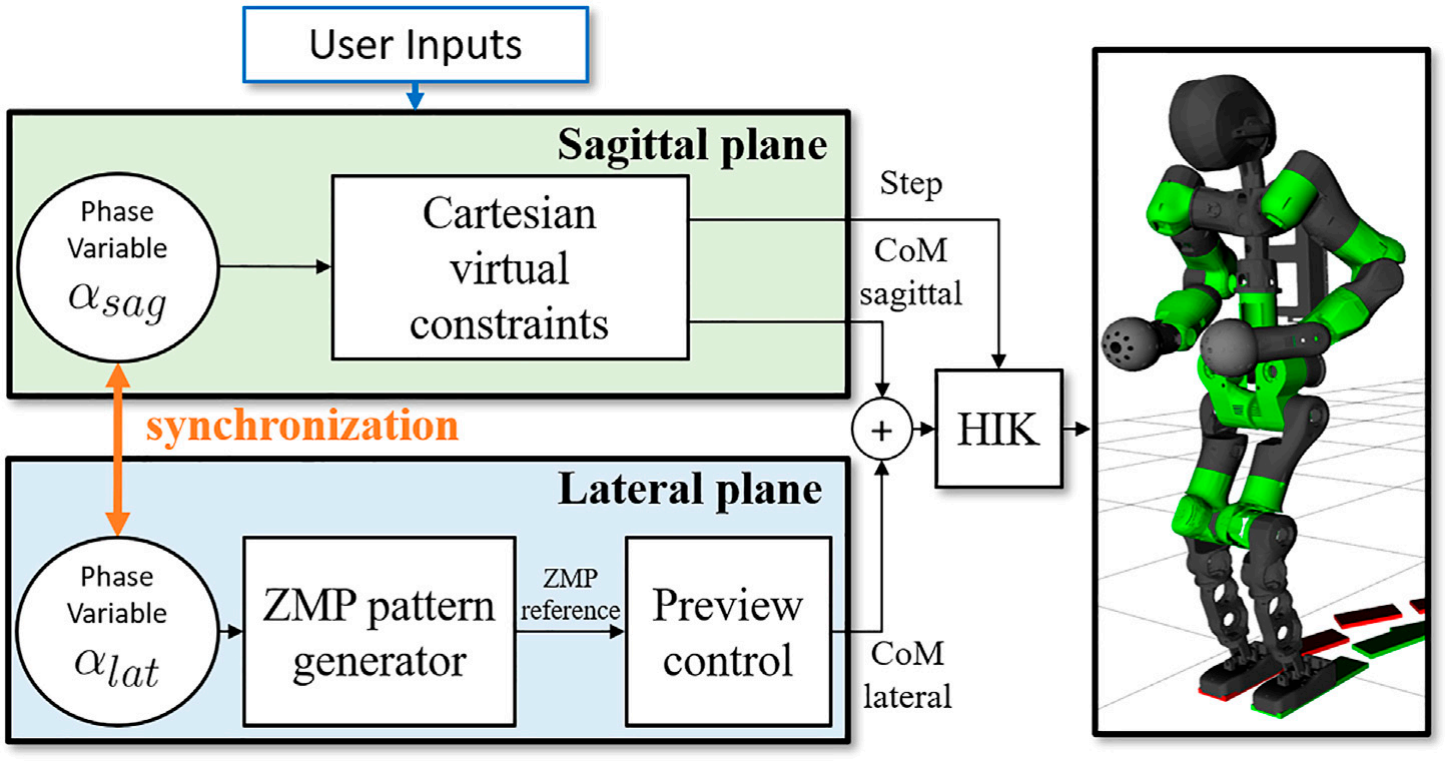
Scheme of the proposed WPG. The sagittal and the lateral components are generated independently using the VC and the PC, based on the online user input received. The phase variables $\theta_{sag}$ and $\theta_{lat}$ govern the evolution of the stepping motion on the two planes, and are synchronized to obtain a feasible gait. The reference trajectories are realized by a hierarchical inverse kinematic (HIK) solver.

The proposed algorithm cyclically generates reference trajectories for the CoM and feet during a single step: their evolution on each plane, from taking off of the swing foot to its landing, is described by the phase variables $\alpha_{sag}$ and $\alpha_{lat}$. The structure of the WPG is shown in Figure 5.

### 6.1 Online Walking Pattern Generator

One step is characterized by a few parameters that can be changed online by the user:

• the maximum inclination $\alpha_{sag}^{max}$ (i.e., stride of the step);
• the steering angle ϕ, which correspond to the angle between the initial and the current direction of the CoM;
• the distance between the feet d.

**FIGURE 6:**
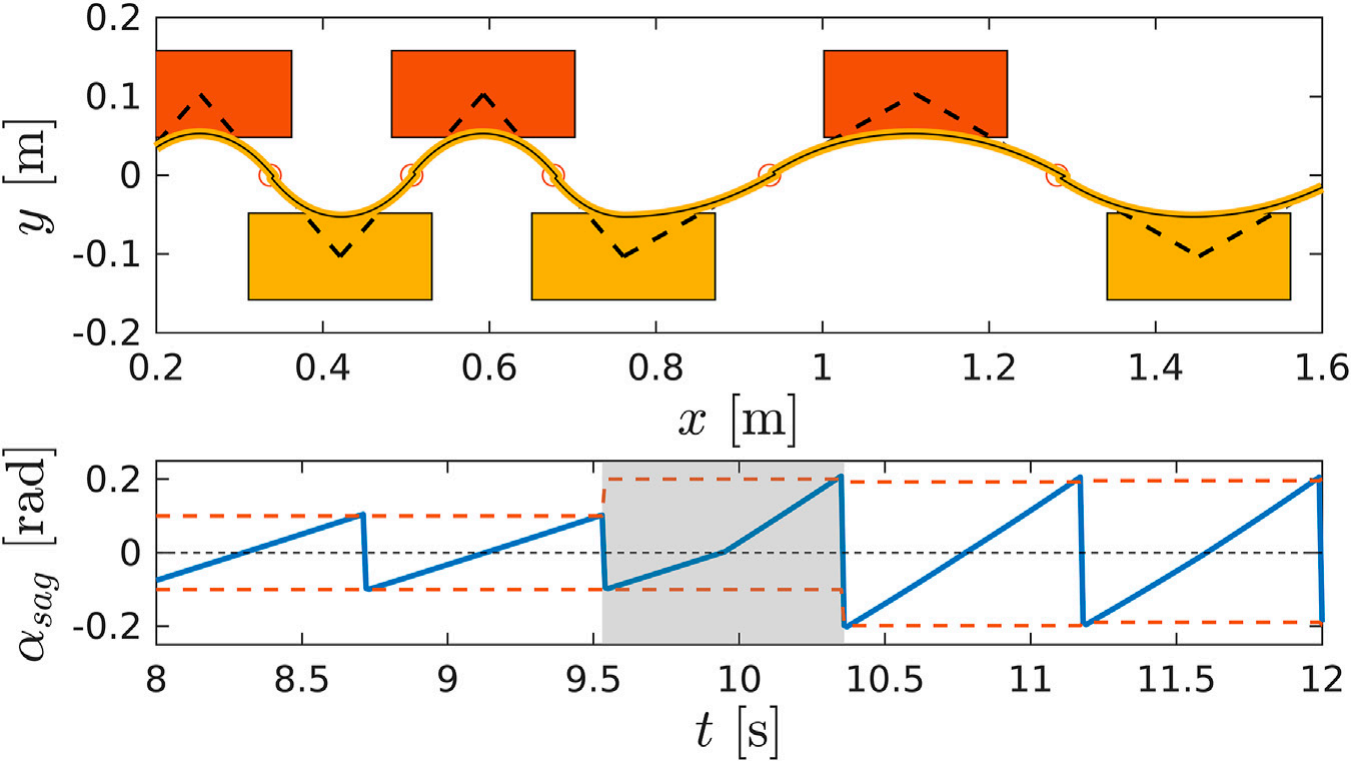
A detail of the WPG changing step stride. Top: walking pattern of the robot changing the length of the step accordingly to the new $\alpha_{sag}^{max}$. Bottom: the trajectory of the phase variable $\alpha_{sag}$. The grey area corresponds to the step where $\alpha_{sag}^{max}$ changes: in the second half of the step the steepness of $\alpha_{sag}$ increases, which corresponds to an increase in the CoM velocity.

The CoM is directly controlled through the phase variable $\alpha_{sag}$: $\alpha_{sag}^{max}$ corresponds to the maximum inclination of the robot, which translates into the length of the step according to the VC in (5): by changing the interval $\left[\alpha_{sag}^{min},\alpha_{sag}^{max}\right]$ a desired stride length can be imposed on the gait. If a new $\alpha_{sag}^{max}$ is issued, the CoM velocity during the step changes accordingly, as shown in Figure 6.

During the stepping motion the *tilt* of the robot $\theta_{1}$ is continuously sensed, and governs the evolution of step. At each impact, the WPG relabels the legs sw and st: the swing leg becomes the stance leg and vice-versa. Moreover, it updates the parameters of the upcoming step:

• updates $\alpha_{sag}^{min}$ with the sensed $\theta_{1}$;
• updates $\alpha_{sag}^{max}$: if no commands were issued by the user, keep the last maximum inclination;
• updates ϕ, if no commands were issued by the user, keep the last heading angle;
• computes the step length, the swing foot goal position and the CoM position as a function of $\alpha_{sag}^{min}$ and $\alpha_{sag}^{max}$.

Notice that, due to the imposed VC, the CoM lies always in the middle of the two feet in the sagittal direction, and when $\alpha_{sag}=0$, the CoM is directly above the stance foot, as shown in Figure 2.

Due to the strategy for synchronization used in the previous WPG in (Ruscelli et al., 2019), the ZMP pattern was generated once during the initialization and the number of steps was decided off-line. As a consequence, it had fixed parameters during execution that could be set only before running the algorithm. The proposed WPG overcomes these limitations, enhancing the versatility of the gait:

• it runs until a stop command is issued;
• it accepts on-line inputs such as step length and feet distance d;
• it allows on-line steering, by issuing the desired heading angle ϕ.

These enhancements entail two other necessary improvements: first, footsteps are no more planned beforehand, but automatically generated on-line during the previous step given the maximum inclination $\alpha_{sag}^{max}$ and the angle of curvature ϕ. Secondly, the preview window is continuously updated with a suitable ZMP reference to accommodate the gait, as shown in Figure 4. The proposed WPG is enclosed in a state machine to manage the walking phases, such as starting and stopping. Finally, the state machine exposes a simple interface for the communication between user and robot.

### 6.2 Whole-Body Inverse-Kinematics

The trajectory are generated on-line by a whole-body IK solver in a SoT fashion (Rocchi et al., 2015; Laurenzi et al., 2019). This allows to run low priority task in the null space of higher priority ones, so as to guarantee the execution of critical tasks above the others. The following SoT was designed for the WPG:

$$\left(\begin{array}{c} (\text{W}T_{\text{LFoot}}{+}^{\text{W}}T_{\text{RFoot}})/\\ (\text{W}T_{\text{CoM}}{+}^{\text{W}}T_{\text{Waist}}^{RPY})/\\ T_{\text{TorsoRoll}}^{\text{Posture}}/\\ T_{\text{Posture}} \end{array}\right)<<(\begin{array}{c} C_{\text{Joint}}\\ \text{Lims} \end{array}+\begin{array}{c} C_{\text{Vel.}}\\ \text{Lims} \end{array})$$ (14)

The / symbol is used to impose *strict* hierarchy among sets of tasks. Among the same level, tasks are listed *via* the + symbol, which impose *soft* hierarchy. The description TℬA define a task of the frame *B* expressed w.r.t. the frame *A*. If *A* is not specified, task $T_{B}$ is expressed in joint space. In the chosen stack,TℬW refers to a generic task *B* w.r.t. the world frame. The Torso task $T_{\begin{array}{c} \text{Posture}\\ \text{TorsoRoll} \end{array}}$ reduces the swing of the robot at each step by keeping the torso perpendicular to the ground. The contact tasks TwLFoot and TRFootW are at the highest priority level. All the other tasks, including the CoM TCoMW, act in the null-space of the contacts to guarantee a consistent solution within the base under-actuation. The pose of the joints not involved in the walking motion is maintained thanks to the Posture task, located in the null space of all the other defined tasks. Finally, the ≪ symbol is used to impose constraints C such as joint limits and joint velocity limits.

## 7 Omnidirectional Walking

**FIGURE 7:**
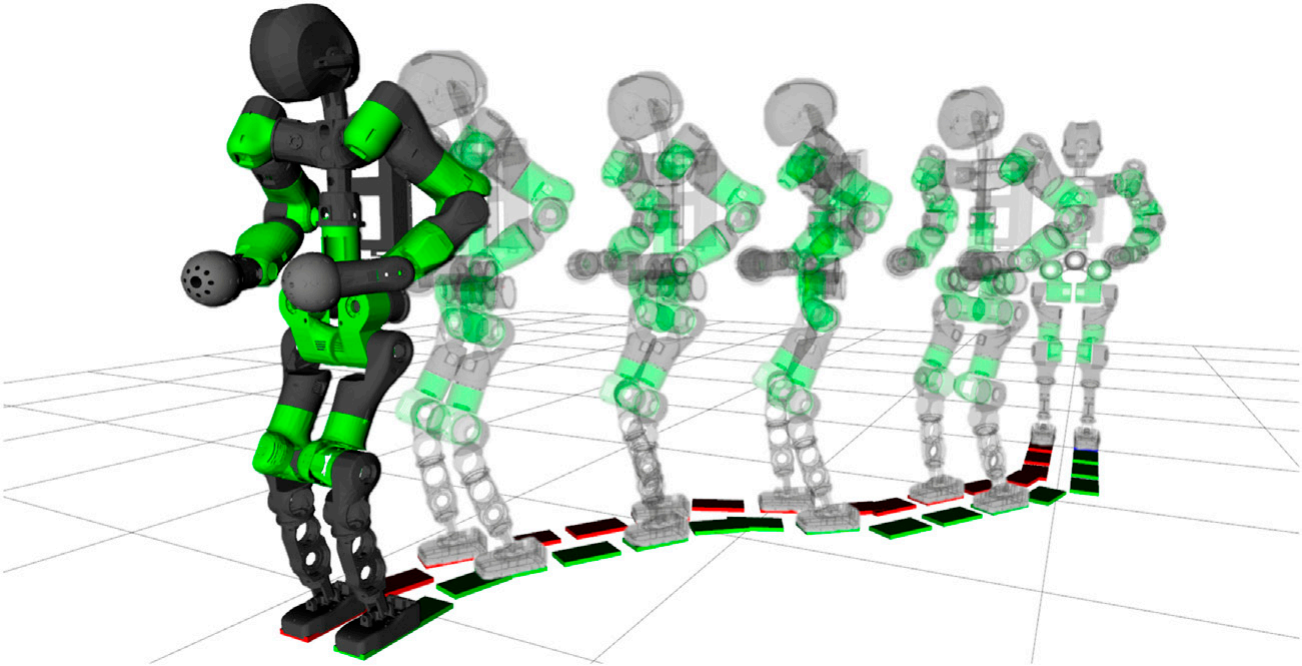
Sequence of frames of the COMAN + performing four steering step, curving two times left of 30° and two times right, steering back in the straight direction.

The previous WPG proposed in (Ruscelli et al., 2019) generates a forward walk without the ability to steer to another direction or walk backwards: due to the inherent decoupling between lateral and sagittal planes, the hybrid WPG is not trivially generalized for omnidirectional walking. In this section, we describe the strategy devised to extend the WPG for steering in any given direction, as shown in Figure 7.

**FIGURE 8:**
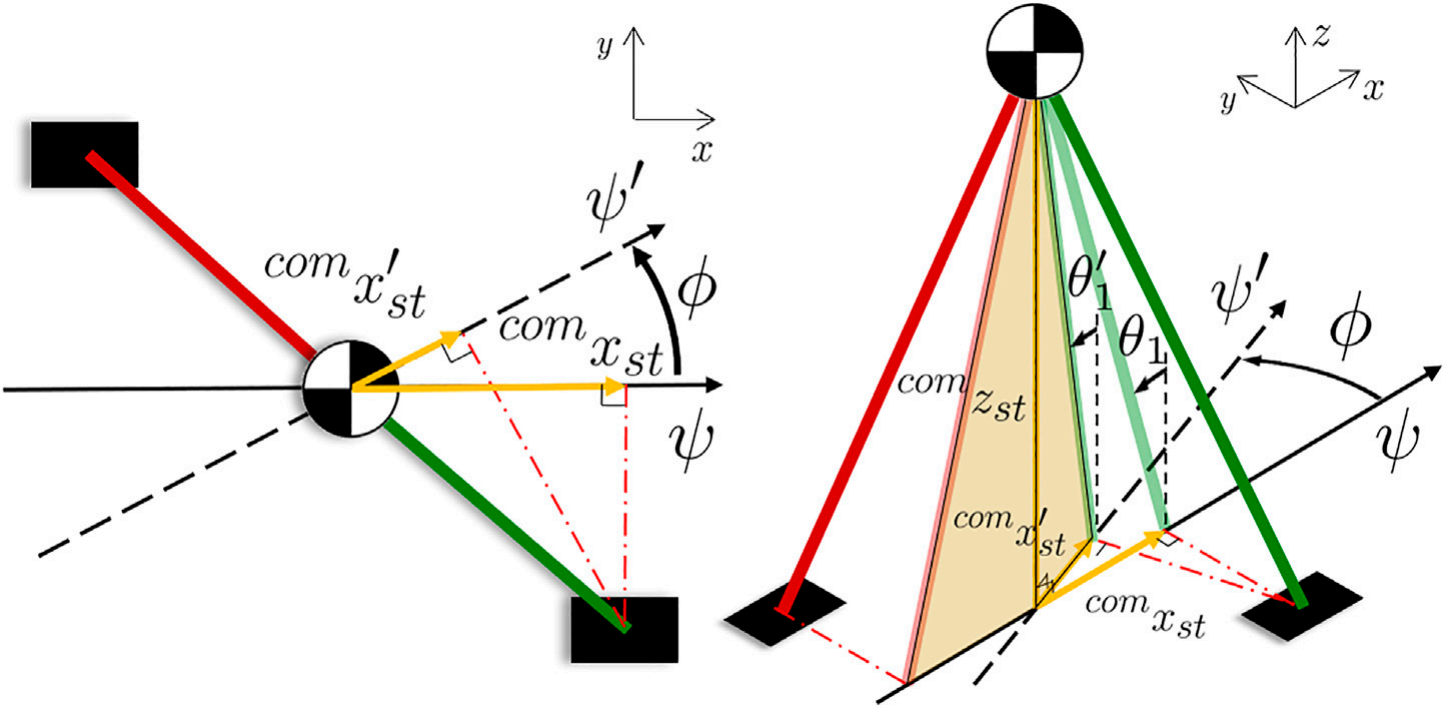
Scheme of the compass model (left: xy-plane view, right: 3D space representation) during a steering step from the heading ψ to ψ'. The sagittal plane rotates of ϕ: the distance between the stance ankle and the CoM decrease, since the ankle position is projected on the new plane. Consequently, the tilt angle $\theta_{1}$ changes and the new $\theta_{1}\text{'}$ must be computed.

For a three-dimensional robot, we define the *tilt angle* $\theta_{1}$ as:

θ1=arctan(xcomstzcomst), (15)

where xcomst and zstcom are the *x* and *z* components of Pstcom∈ℝ3, i.e., the *stance foot* ankle w.r.t. a reference frame centered at the CoM and oriented with the x-axis aligned to the direction of motion. When a steering angle ϕ is issued, a steering step is performed to change the direction of motion from the heading angle ψ to the new one ψ′=ψ+ϕ, and the reference frame at the CoM is rotated by ϕ along the z-axis. The position of the stance ankle is recomputed according to the rotation:

Pstcom′=R(ϕ)compst, (16)

where $R\left(\phi \right)\in {\mathbb{R}}^{3\times 3}$ is the rotation matrix representing a rotation by ϕ on the horizontal plane (Figure 8). This cause the *tilt angle* $\theta_{1}$ to change during the steering motion: once the rotation (16) is applied, the new $\theta_{1}\prime$ is computed at the beginning of the steering step by applying (15).

The walking resumes with the new $\theta_{1}\prime$ as the phase variable $\alpha^{min}$, as described in Section 6. The algorithm is designed to keep the distance between the legs *d* constant after the change of heading. To compute the parameters of the steering step, the following procedure is carried out:

• the goal pose of the *swing foot* Tswgoalst is computed w. r.t the *stance foot* given the current heading ψ and the phase variable $\alpha^{max}$;
• Tswgoalst is rotated by ϕ along the z-axis. The distance between the feet *d* is constant;
• the goal position of the CoM $p_{com}^{goal}$ lies in the middle of the *stance foot* and the new *swing foot* position;
• set $\alpha^{max}$ according to (3), so that the CoM position reaches $p_{com}^{goal}$ at the end of the step;
• set $\alpha^{min}$, which corresponds to the new *tilt angle* $\theta_{1}\prime$ computed using (15) and 16.

the CoM moves forward in the sagittal plane until α reaches $\alpha^{max}$ and the foot impacts the ground. At the impact, the new angle of heading ψ is updated with the angle of steering ϕ, and the walk resumes in the new direction.

**FIGURE 9:**
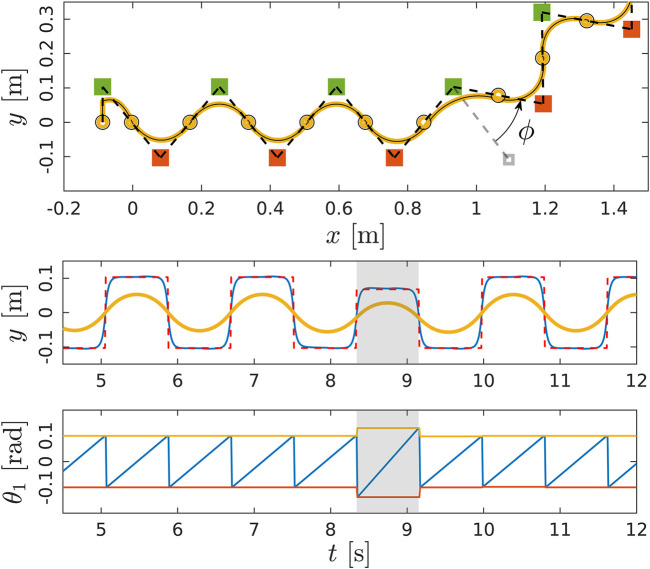
The WPG steers the robot of an angle ϕ. Top: walking pattern of the robot, including the pose of the left (in green) and right (in red) sole and the CoM trajectory (in yellow). Middle: ZMP reference for the preview control. Bottom: the evolution of the tilt angle $\theta_{1}$. The grey area highlights the steering step.

The distance *d* between the right and the left sole in the lateral plane and the tilt in the sagittal plane $\alpha^{max}$ (which corresponds to the step stride) are kept constant before and after the curve. During the steering, due to the rotation of the reference frame, both *d* and $\alpha^{max}$ are adjusted to generate the desired step: the ZMP pattern fed to the preview control and the phase variable are updated as shown in Figure 9.

## 8 Experiments and Discussion

The walking pattern generator was tested in simulation using Gazebo and validated on COMAN+, a humanoid robot developed at Istituto Italiano di Tecnologia. COMAN + has 28 DoFs, weights 70 kg and is 1.7 m tall. A particular four-bar mechanism controls the pitch and the roll at the ankle level of the robot (Ruscelli et al., 2018). The WPG prescribes position references that are tracked on the robot by a whole-body IK framework named CartesI/O (Laurenzi et al., 2019) based on OpenSoT Rocchi et al. (2015), which allows to specify the desired SoT solved by quadratic programming (QP) optimization. COMAN+ is powered by the XBotCore real-time software architecture (Muratore et al., 2017). A ROS pipeline allows communication between user WPG and lower level. The WPG runs at 100 Hz in a ROS node and exposes a simple ROS interface for issuing commands to the robot.

### 8.1 Experiments

**FIGURE 10:**
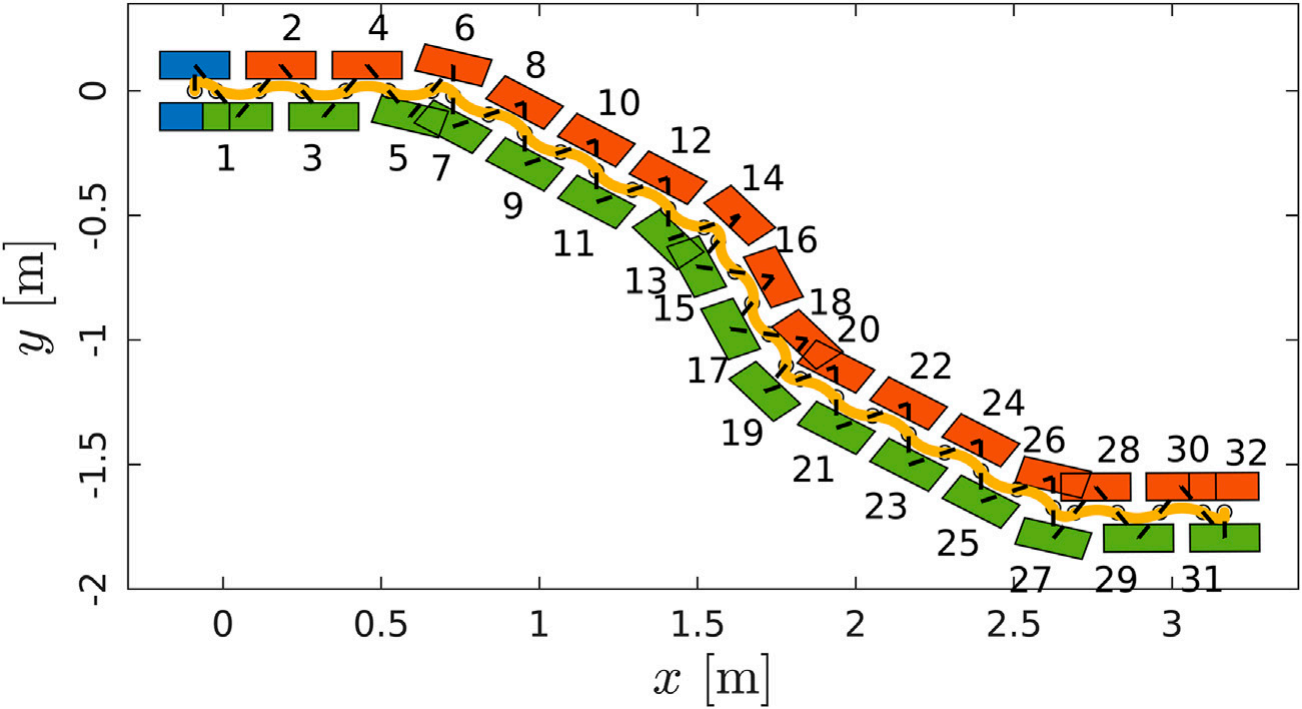
Walking pattern consisting of the CoM trajectory (in yellow) and the footsteps (initial pose in blue, left and right soles in red and green respectively). COMAN + walks 32 steps with a 0.28 m step stride, 0.05 cm step clearance and 0.4 s step duration. The heading changes of $\phi =-30^{\circ }$ at step 5 and 13 and of $\phi =30^{\circ }$ at step 19 and 27.

**FIGURE 11:**
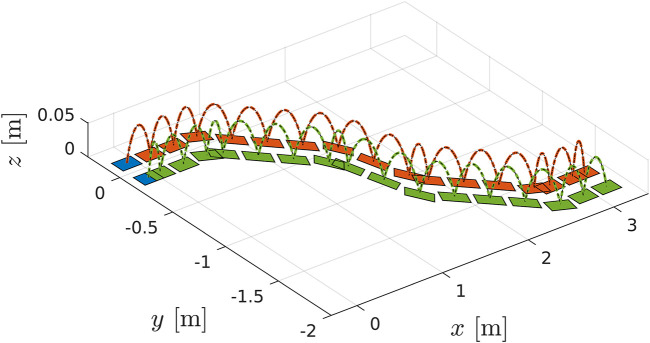
Detail of the swinging foot trajectory with a 0.05 m step clearance.

**FIGURE 12:**
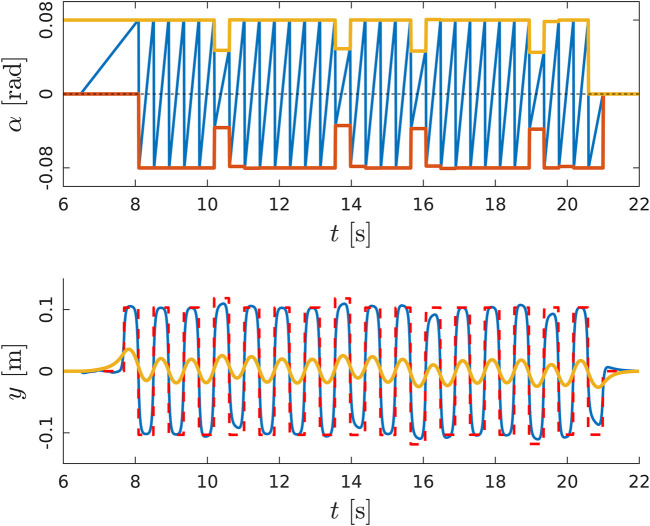
Top: evolution of the phase variable $\alpha_{sag}$ (in blue) in the sagittal plane during a walk in simulation, increasing at each cycle from $\alpha_{sag}^{min}$ (in red) to $\alpha_{sag}^{max}$ (in yellow). Bottom: the ZMP input reference of the PC (in red) and the tracked ZMP (in blue). The output of the PC is the CoM trajectory (in yellow) commanded to the robot.Sequence of frames of COMAN + walking 32 steps in simulation while changing the heading direction.

**FIGURE 13:**
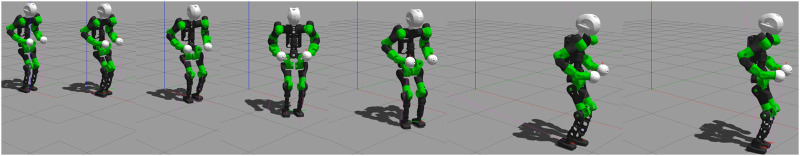
The output of the PC is the CoM trajectory (in yellow) commanded to the robot.Sequence of frames of COMAN + walking 32 steps in simulation while changing the heading direction.Sequence of frames of COMAN + walking 10 step and steering of 10° at step 3 according to the issued command.

**FIGURE 14:**
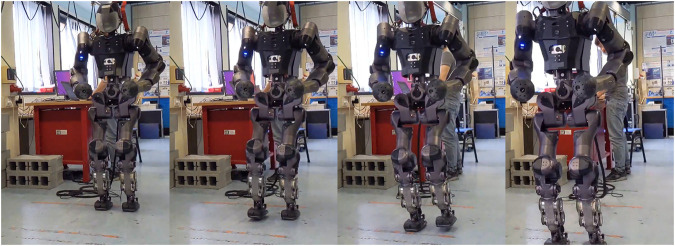
Sequence of frames of COMAN + walking 10 step and steering of $10^{\circ }$ at step 3 according to the issued command.

A set of experiments was carried out both in simulation and on real hardware for the robot COMAN+, in order to validate the WPG. We detail here one meaningful example in simulation. COMAN + walks 32 steps, with a 0.05 m step clearance, a 0.4 s step duration and a maximum tilt of 0.08 rad, which corresponds to a 0.28 m step stride. Figures 10,11 shows the projection of the walking pattern on the x-y plane and the evolution of the soles in space, respectively: the CoM oscillates laterally between the legs, from the stance towards the swinging foot. The faster the gait velocity, the smaller the lateral swings of the CoM. During step 5 and 13 a new $\phi =-30^{\circ }$ is issued, while at step 19 and 27 the robot steers back of $\phi =30^{\circ }$ to the straight direction. The WPG can steer up to 30 deg in a single step. The maximum steering angle is limited by the hybrid nature of the proposed architecture: during the steering step the preview control only works in the previous lateral direction, and a component of the lateral CoM trajectory will not be guaranteed to belong to the support polygon. The maximum speed of the robot is 1.25 m/s in simulation when moving forward, while the maximum step length change is 0.4 m (from 0.1 to 0.5 m). Figure 12 depicts the time evolution of the ZMP reference in the lateral plane and the phase variable $\theta_{sag}$ along the sagittal direction. In particular, the preview controller tracks the reference ZMP, which is continuously updated during the stepping motion, and outputs the lateral CoM trajectory. The phase variable $\theta_{sag}$ constrains the evolution of the step, spanning from $\theta_{sag}^{min}$ to $\theta_{sag}^{max}$ at each cycle after an impact occurs, and it is synchronized with the evolution of the phase variable $\alpha_{lat}$ in the lateral plane. A sequence of frames of the experiment is depicted in Figure 13. The WPG was deployed on real hardware to assess its effectiveness (Figure 14). The experiment consisted of a 10 step walk with 0.23 m stride length. The selected step clearance was 0.05 m and the step duration of 0.4 s. A steering angle of $10^{\circ }$ was issued at step 3. While the algorithm generates feasible trajectories for the desired gait, the WPG does not guarantee a stable walking: the reference trajectories are open-loop, since no stabilization controller is added to react against an unwanted loss of balance or reject external disturbances. Videos of the experiments both in simulation and on the real robot can be found at https://www.youtube.com/playlist?list=PL7c1ZKncPan7yphDxvZDtaqzmgCRtpT5-.

### 8.2 Discussion

The proposed WPG is a lightweight algorithm that can be easily deployed on real hardware. The classical implementation of VC entails joint-trajectory optimization to find energy-efficient gaits. Often the optimization is run offline individually for each desired gait that corresponds to one optimal orbit (or a family of similar orbits). Providing a walker with different gaits usually requires a library of optimal parameters collected beforehand. This leaves less margin to tune a walking pattern or change it on the fly, hindering the versatility of the method. Furthermore, the VC usually binds joint variables, making it heavily dependent on the mechanism kinematics. Instead, we treat the VC as Cartesian tasks in a SoT and we design template trajectories that can be tuned and realized on line by the whole-body IK solver. This allows a light and flexible tool for stepping that only depends on a simple template model and a few step parameters, such as length, duration and clearance of a step. The new WPG also allows to modify the heading angle and the distance between feet. According to the belief that a more dynamic approach can be used along the sagittal plane while a more conservative one can be exploited laterally, in our strategy the CoP is guaranteed to lay inside the support polygon only in the lateral direction, while in the sagittal one it can move freely. On the other hand, our strategy has a higher energy consumption with respect to the classical approach, because it doesn’t exploit the natural dynamic of the system during a step. In fact, the trajectory of the CoM is fully controlled. Right now the commanded trajectory is linear, but a possible solution is finding an optimized trajectory of the CoM to minimize a desired cost function. Notice that the CoM trajectory during a step is directly related to the *tilt* of the robot, which corresponds to the phase variable. Hence, modifying the CoM trajectory amount to changing the behaviour of the stepping. Another limitation of the strategy is the amount of modification that the gait can sustain in one single step: being a cyclic motion, abrupt changes in the direction of movement or in the step length will result in the robot losing balance. Since the work was only focused in the development of a WPG, a feedback term of the measured state of the robot was not included: hence no information from the actual robot is exploited in the algorithm.

The major downside of this choice is reducing the dynamic component of the walking motion in the sagittal direction. While part of the walking can be controlled directly imposing a CoM trajectory, the portion of the gait which entails a “controlled” fall can be exploited only given a proper state estimation: sensing the evolution of the tilt angle $\theta_{1}$ injects into the system the free-fall dynamics of the stance leg, used by a continuous feedback controller to restrict the stepping motion on a periodic orbit. Finally, while simulation experiments show successful results, their hardware counterpart is not as satisfying: this discrepancy is mainly due the lack of a stabilization effect that becomes necessary for real robot experiments. While the open-loop WPG plans feasible trajectories, as discussed in Appendix and demonstrated in simulation where the factors acting as disturbances can be limited, a control layer is essential when deploying the WPG in a real-word scenario. Nonetheless, including hardware results highlights how this strategy is viable, displaying promising results towards a WPG that integrates a planning and a control layer.

## 9 Conclusions and Future Work

The proposed walking pattern generator, due to its hybrid nature, is not trivially generalizable for omnidirectional gaits: in order to do so, we improved the synchronization of the lateral and sagittal planes, enabling on-line modifications to the walking gait. The resulting algorithm is a lightweight, omnidirectional WPG which generates feasible whole-body trajectories according to a few user-parameters. Besides off-line specifications such as duration, length and stride of the desired step, the proposed WPG allows on-line commands to change heading angle, step length and feet distance. The sagittal motion of the CoM is directly commanded as a simple linear trajectory. In future works, we will use optimization techniques to select a trajectory that minimizes a desired function such as power consumption. Simulation experiments demonstrate the effectiveness of our strategy: the robot walks and changes the nominal gait according to user-inputs without falling. Furthermore, preliminary experiments on the humanoid COMAN + shows how the proposed hybrid WPG is promising, successfully allowing the robot to change its heading direction. However, without a stabilization controller, any external disturbance (e.g., uneven or sloped terrain) is enough to make the robot lose balance after a few steps.

Similarly, the maximum steering angle that can be issued on the real robot is limited, as the weaknesses highlighted in the straight walk are sharpened when changing direction.

In future works, we will close the loop with the external environment by implementing stabilization techniques to add robustness to the gait.

## Data Availability

The raw data supporting the conclusion of this article will be made available by the authors, without undue reservation.

## Footnotes

1 *The source code of the Hybrid WPG can be found at https://github.com/ADVRHumanoids/limit_cycle_walking

2 From here on, we drop the notation of *x* in α(x) for simplicity.

## 10 APPENDIX

The WPG is one of the building components of a full-fledged locomotion framework. Its goal is to compute suitable trajectories, such that the overall motion of the robot is dynamically consistent under ideal conditions (absence of external disturbances, no discrepancies between the model and the robot, perfect tracking of the computed references). This appendix is devoted to the discussion and the evaluation of the aforementioned aspect for the devised hybrid framework. In Subsection 3.3 we outline the role of the *tilt angle* $\theta_{1}$ in the overall robot motion and the choice to directly control it exploiting the full-actuation of COMAN+. Differently from the classical theory of HZD, where the tilt angle is sensed from the robot and used to compute a swing leg trajectory, given a set of VC that encode a walking pattern guaranteeing stability in the form of a limit cycle. In Westervelt et al. (2007) it is shown how this can be achieved by input-output linearizing the swing phase dynamics and imposing a desired dynamic response on the outputs. Given a set of virtual constraints in the form of 1, and a continuous feedback controller that drives to zero the output *z* in a finite time, the non-controlled evolution of $\theta_{1}$ injects in the system the free-fall dynamics of the stance leg, while the controller guarantees that the evolution of the swing phase restricts the stepping motion on a periodic orbit. Conversely, in our approach $\theta_{1}$ is explicitly controlled. First, due to the lack of a base estimation on our robot COMAN+, the tilt angle could not be easily sensed and inserted in a feedback loop. Secondly, dealing with a fully controllable system implies some advantages, as it can be easily regulated to impose desired behaviours. Without discarding the possibility to add a feedback controller to exploit the natural dynamics of the robot in the forward motion, this framework focus on the planning of the CoM of the robot as a fully controllable variable. In particular, it is commanded to move with a constant velocity throughout the whole stepping motion:

• *Stability of the motion in the sagittal plane*: since the CoM velocity is constant, the ZMP coincides with the CoM during the motion. As long as the CoM dwells inside the support polygon, the ZMP does the same. When the gait is initiated, the WPG applies a constant CoM acceleration to reach the desired constant velocity. Indeed, the initial acceleration cannot exceed a threshold, as the ZMP would leave the support polygon of the robot. We will briefly analyse the initial step, introducing some relevant quantities. Let *l* be the length of the foot, from heel to toes. The initial position of the CoM, $x_{\text{com}}$, rests in the center of the sole. Given the ZMP equation in the sagittal plan,

$$x_{\text{zmp}}=x_{\text{com}}-\frac{h_{\text{com}}}{g}{\ddot{x}}_{\text{com}},$$ (17)

one must guarantee that the ZMP stays in the support polygon at all times. Given the constant acceleration, it is enough to prove it for the instant the acceleration is applied, which amounts to say that the ZMP instantaneously doesn’t leave the support polygon:

$$x_{\text{com}}-l/2<x_{\text{zmp}}$$ (18)

which, using (17), yields the maximum acceleration allowed:

$${\ddot{x}}_{\text{com}}<\frac{gl}{2h_{\text{com}}}$$ (19)

The same reasoning can be used for the deceleration of the CoM at the end of the gait, which brings to a stop the humanoid motion.

• *Stability of the motion in the lateral plane*: the preview control governing the lateral swing is made dependent on time and not on space: in particular, the advancement of the preview window is directly dependent on the phase variable in the sagittal plane. If the phase variable evolution (from its minimum $\alpha_{sag}^{min}$ to its maximum $\alpha_{sag}^{max}$) is not constant during one cycle (i.e. one stepping motion), the sliding of the preview window slows down or accelerates, and the assumptions for a dynamically consistent motion would be lost. However, since the phase variable in the sagittal plane $\alpha_{sag}$ is directly controlled, we can command a constant increment w.r.t. time, which corresponds to a constant sliding of the preview window, guaranteeing stability of the swing motion along the lateral plane.

Nonetheless, this does not mean that $\alpha_{sag}$ is restricted to increase constantly: as described in Section 6, one of the improvement of the paper consists in allowing the preview window to be continuously updated with a suitable ZMP reference to accommodate the gait. In the case of an acceleration or a deceleration of the phase variable $\alpha_{sag}$, the CoM accelerate or a slows down, since $\alpha_{sag}$ is the tilting angle between the ankle and the CoM. Hence, knowing the lateral CoM state, a feasible position of the next foot can be obtained using the Capture Point (Pratt et al., 2006):

$$y_{cp}=y_{com}+\frac{{\ddot{y}}_{com}}{\omega }$$ (20)

and the ZMP preview window recomputed accordingly, setting the ZMP on the instantaneous CP.
